# RIG-I Detects Triphosphorylated RNA of *Listeria monocytogenes* during Infection in Non-Immune Cells

**DOI:** 10.1371/journal.pone.0062872

**Published:** 2013-04-30

**Authors:** Cristina Amparo Hagmann, Anna Maria Herzner, Zeinab Abdullah, Thomas Zillinger, Christopher Jakobs, Christine Schuberth, Christoph Coch, Paul G. Higgins, Hilmar Wisplinghoff, Winfried Barchet, Veit Hornung, Gunther Hartmann, Martin Schlee

**Affiliations:** 1 Institute for Clinical Chemistry and Clinical Pharmacology, University Hospital Bonn, University of Bonn, Bonn, Germany; 2 Institutes of Molecular Medicine and Experimental Immunology, University of Bonn, Bonn, Germany; 3 Institute of Medical Microbiology, Immunology and Hygiene, University of Cologne, Cologne, Germany; 4 German Center for Infection Research, Cologne-Bonn, Germany; Institut de Pharmacologie et de Biologie Structurale, France

## Abstract

The innate immune system senses pathogens by pattern recognition receptors in different cell compartments. In the endosome, bacteria are generally recognized by TLRs; facultative intracellular bacteria such as *Listeria*, however, can escape the endosome. Once in the cytosol, they become accessible to cytosolic pattern recognition receptors, which recognize components of the bacterial cell wall, metabolites or bacterial nucleic acids and initiate an immune response in the host cell. Current knowledge has been focused on the type I IFN response to *Listeria* DNA or *Listeria*-derived second messenger c-di-AMP via the signaling adaptor STING. Our study focused on the recognition of *Listeria* RNA in the cytosol. With the aid of a novel labeling technique, we have been able to visualize immediate cytosolic delivery of Listeria RNA upon infection. Infection with *Listeria* as well as transfection of bacterial RNA induced a type-I-IFN response in human monocytes, epithelial cells or hepatocytes. However, in contrast to monocytes, the type-I-IFN response of epithelial cells and hepatocytes was not triggered by bacterial DNA, indicating a STING-independent *Listeria* recognition pathway. RIG-I and MAVS knock-down resulted in abolishment of the IFN response in epithelial cells, but the IFN response in monocytic cells remained unaffected. By contrast, knockdown of STING in monocytic cells reduced cytosolic *Listeria-*mediated type-I-IFN induction. Our results show that detection of *Listeria* RNA by RIG-I represents a non-redundant cytosolic immunorecognition pathway in non-immune cells lacking a functional STING dependent signaling pathway.

## Introduction

Multicellular organisms evolved efficient host-defense mechanisms to sense invading pathogens such as bacteria and viruses in order to block their replication and spread. Components of the bacterial cell wall and conserved bacterial proteins are recognized by germ line encoded Toll-like receptors (TLRs) on the surface of cells and by receptors of the nucleotide-binding domain and leucine-rich repeat containing gene family (NLRA,B,C,P,X) in the cytosol [1]. Recognition of nucleic acids plays a major role in immune responses during viral infection. The pathogenicity of foreign genetic material is either recognized through its location, specific structural features or modification of nucleic acids. Nucleic acids are recognized by TLRs in the endosome. TLR3 detects double stranded RNA. TLR7 and TLR8 detect double and single stranded RNA respectively, with a preference for certain sequence motifs (reviewed in [2]). TLR9 is activated by DNA containing unmethylated CpG motifs [3], [4], [5]. In the cytosol, the helicase MDA5 [6] recognizes long double stranded RNA with additional structural requirements as yet not well defined [7], [8] and the helicase RIG-I [6] binds to and is activated by 5′triphosphorylated double stranded RNA [9], [10], [11], [12], which is present either in viral genomic RNA or is formed during viral replication. Two independent studies described a TLR9-independent pathway of cytosolic recognition of double stranded DNA leading to type I IFN induction [13], [14]. So far, numerous candidate cytosolic DNA receptors including DAI, DDX41, IFI16, LRRFIP1, Ku70, DHX36, DHX9 and DNA-PK have been suggested [15]. All studies agree on the fact that the mitochondrial adaptor protein STING (also known as MITA) downstream of the putative DNA receptor is essential for sensing cytosolic DNA [16], [17]. A recent study by Sun et al. identified *Cyclic GMP-AMP Synthase* (cGAS) as the so far most convincing DNA recognizing candidate receptor [18]. Upon recognition of DNA, cGAS synthesize the second messenger *Cyclic GMP-AMP (cGAMP)* which activates STING [18], [19].

Several groups [20], [21], [22] identified AIM2 to be a cytosolic dsDNA sensing receptor, which activates caspase 1, leading to the release of IL-1. While the role of AIM2 in the activation of caspase-1 is non-redundant, AIM2 is not involved in the activation of transcription factors. Recent work demonstrated that AT-rich DNA can be sensed by an indirect recognition mechanism. It was demonstrated that the endogenous RNA polymerase III (pol III) uses AT-rich DNA (poly(dA-dT)) as a template, leading to generation of 5′triphosphorylated poly AU-RNA, which forms an dsRNA and therefore represents a strong RIG-I ligand [11], [23], [24]. Due to the ubiquitous expression of RIG-I, poly(dA-dT)-mediated type I IFN induction appears to occur in all cell types analyzed so far. However, this pol III/RIG-I dependent pathway for type I IFN induction is not activated upon transfection of mixed DNA sequences lacking AT rich regions such as plasmid DNA or PCR products. In the human system only certain immune cells with an intact STING-dependent DNA sensing pathway, e.g. monocytic cells, are able to induce type I IFN by PCR products or plasmid DNA.

IFN induction by RNA from RNA viruses has been explored and understood far better than type I IFN induction by DNA. Binding of RIG-I and MDA5 to the mitochondrial adaptor molecule MAVS (also known as IPS1, Cardif or VISA [25], [26], [27], [28]) leads to the binding and the activation of IRF3, which induces type I IFN expression. MAVS is essential for RIG-I and MDA5 signaling, but not for RIG-I independent ( = STING dependent) pathways of dsDNA mediated type I IFN induction [29].

Bacteria trigger IFN-β responses through stimulation of TLR4 on the cell surface or TLR9 in endosomes. Investigations in the labs of Decker and Portnoy suggested that TLR-independent pathways exist, which lead to the induction of type I IFN in mouse macrophages infected with *Listeria monocytogenes* [30], [31]. Interestingly, type I IFN induction depended on cytosolic localization of the bacteria [30], [31] but was shown to be NOD2-independent [32]. Later on, the requirement of MAVS and, consequently, MDA5 or RIG-I in type I IFN induction was excluded in murine bone marrow-derived or peritoneal macrophages and MEFs [29], [33]. Stetson and Medzhitov [13] observed that DNA represents the type I IFN inducing agent in the lysate of *Listeria monocytogenes* when transfected into murine monocytes. From their experiments, they concluded that intracellular bacteria (*L. monocytogenes and Legionella pneumophila*) with cytosolic access or cytosolic contact, induce a type I IFN response upon recognition of bacterial DNA in the cytosol. Moreover, cyclic diadenosine monophosphate (c-di-AMP), a metabolite of *L. monocytogenes* similar to the endogenous second messenger *cGAMP* was found to induce type I IFN induction directly via STING [34], [35]. The fact that the DNA-sensing AIM2 inflammasome [20], [21], [22] is involved in *Listeria-*induced caspase 1 activation [36], [37], [38], clearly supports the contribution of released bacterial DNA to the immune response.

Here we hypothesized that, like DNA, bacterial RNA can enter the cytosol, where it is recognized by cytosolic RNA receptors, for example RIG-I-like helicases. Indeed, in contrast to eukaryotic mRNA, *bacterial* mRNA is not capped but contains 15% 5′triphosphorylated RNA [39], which would render bacterial RNA an ideal PAMP due to its ability to activate RIG-I. In this study, we used the bacterium *L. monocytogenes* as a model organism for intracellular bacteria-host interaction. *L. monocytogenes* is an opportunistic bacterium responsible for human food-borne infections leading to meningitis and miscarriages. After crossing the intestinal barrier it enters lymph nodes, spleen and liver. In immunocompromised individuals, bacterial multiplication can occur in hepatocytes with further release of the bacteria into the blood and spread to the brain and the placenta (reviewed in [40]). Crossing the host barriers involves bacterial invasion and survival within a large variety of nonphagocytic cells [41]. Internalins (InlA and InlB) allow invasion via E-cadherin on the surface of epithelial cells and via hepatocyte growth factor receptor (MET), which is expressed on a wide range of cells [41]. With the current study, we provide direct evidence that RNA isolated from bacteria induces type I IFN in a 5′ phosphorylation-dependent manner. Using an advanced RNA labeling technique, we were able to show that RNA from *L. monocytogenes* translocates to the cytosol of the host cell during infection. This RNA sensing pathway induced both type I IFN and CXCL10 during infection with *L. monocytogenes* in non-monocytic cells, including hepatocytes and colon epithelial cells. Using RNAi we revealed that RIG-I is crucial for *L. monocytogenes*-induced type I IFN and CXCL10 induction in cell types, such as non-immune cells, without a functional STING-dependent immune response, as indicated by the absence of a direct sensing mechanism for cytosolic DNA.

## Results

### Bacterial RNA is Recognized by Human Monocytes by a TLR-independent but RNA 5′-phosphate Dependent Pathway

**Figure 1:**
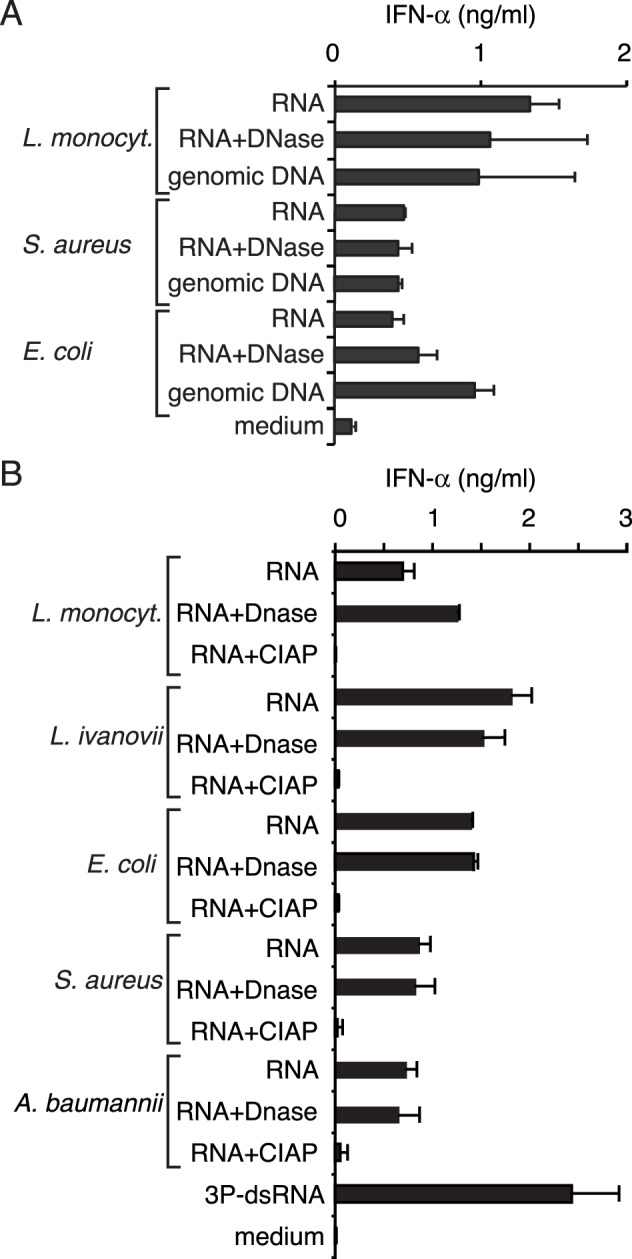
Bacterial RNA is recognized by human monocytes in a TLR-independent and 5′phosphorylation-dependent pathway. Human PBMC were preincubated with chloroquine and transfected with indicated nucleic acids. IFN-α production was analyzed 24 hours after stimulation. Error bars represent s.d. A: The IFN-α-inducing activity of bacterial RNA (untreated or DNase treated) and DNA of *L. monocytogenes*, *Staphylococcus aureus* and *Escherichia coli* was analyzed. B: The IFN-α-inducing activity of RNAs from *L. monocytogenes*, *L. ivanovii*, *E. coli, S. aureus* and *Acinetobacter baumannii,* treated with DNase or calf intestine alkaline phosphatase (CIAP), were compared.

Initially, we evaluated whether bacterial DNA and RNA isolated from extracellular and intracellular bacteria can trigger a TLR-independent type I IFN response. For this purpose, PBMCs were preincubated with chloroquine to block endosomal TLRs (TLR7, TLR8 and TLR9) and were then transfected with bacterial DNA (bacDNA) or bacterial RNA (bacRNA) extracted from the indicated bacteria (Fig. 1A). Chloroquine suppresses endosomal TLR-activity [46] as monitored by CpG ODN transfection (Fig. S1). DNase I-treated bacRNA still induced type I IFN to the same degree as non-treated bacRNA, indicating that DNA contamination did not account for the stimulatory activity (Fig. 1A). As shown previously, triphosphates at the 5′ end are critical for RIG-I-mediated recognition of RNA [10]. To address the involvement of RIG-I in bacRNA recognition, RNA was treated with alkaline phosphatase to remove triphosphates from the 5′ ends (Fig. 1B). Indeed, dephosphorylation of bacRNA diminished IFN-α-inducing activity, suggesting a triphosphate-dependent activation pathway (Fig. 1B). This activation pattern was similar for all analyzed extracellular or facultative intracellular bacteria including E. *coli* (extracellular), *L. monocytogenes* (facultative intracellular), *Staphylococcus aureus* (facultative intracellular, [47]) and *Acinetobacter baumannii* (facultative intracellular [48]) (Fig. 1B). Altogether, these data indicate that the RNA of all tested bacteria activate a triphosphate dependent, TLR independent, type I IFN-inducing pathway when transfected into cells.

### RNA of *Listeria monocytogenes* has Access to the Cytosol of the Host Cell during Infection

**Figure 2:**
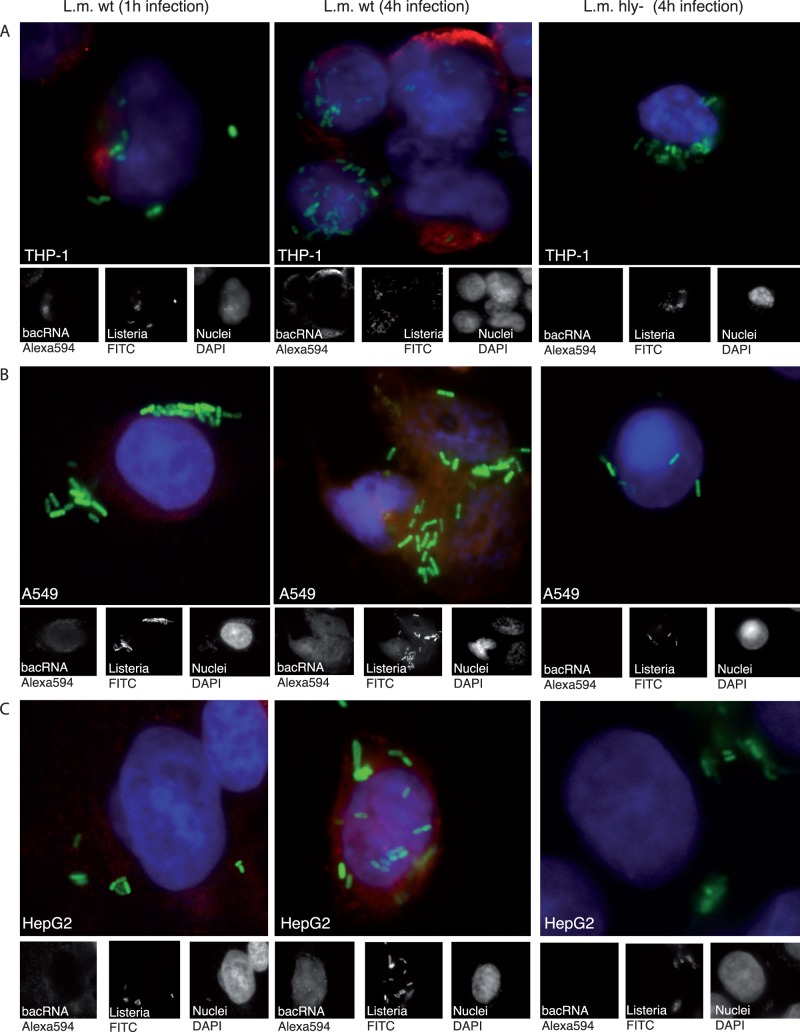
RNA of *L. monocytogenes* has access to the cytosol of the host cell during infection. THP-1, A549 and HepG2 were infected with FITC-tagged and EU-labeled wt and hly^-^ *L. monocytogenes* for the indicated duration. Cells were then fixed, stained with Alexa594-azide and counterstained with DAPI. Left column, wt *L. monocytogenes* infection 1 hr. Middle column, wt *L. monocytogenes* infection 4 hrs. Right column, hly^-^ *L. monocytogenes* infection 4 hrs. A: THP-1 cells. B: A549 cells. C: HepG2 cells. As determined by counting of single bacteria in cells (50 cells per slide were counted) the average bacterial load was 9(wt) and 4(hly^-^) bacteria per cell for THP-1 cells, 6(wt) and 4(hly^-^) bacteria per cell for A549 cells and 6(wt) and 5(hly^-^) bacteria per cell for HepG2 cells, one representative experiment out of two is shown. Whole *L. monocytogenes* are labeled green with FITC and RNA is visible as red fluorescence (Alexa594), nuclei are stained by DAPI (blue).

To assess the relevance of our findings in the host-pathogen interaction during infection we investigated whether bacRNA is able to reach RIG-I localized in the cytosol of the host cell. The bacterium *L. monocytogenes* is a well-characterized model organism for studying intracellular bacteria-host interaction. In particular, it is able to escape the endosome and migrate into the cytosol of macrophages, human (not murine) epithelial cells [49], [50] or hepatocytes [51], a process mediated by the bacterial pore-forming toxin “listeriolysin” (LLO, encoded by the *hly* gene). In the following experiments, we selected cell lines that are derived from tissues or cell types involved in *L. monocytogenes* pathogenesis *in vivo*. THP-1 is an acute monocytic leukemia cell line similar to human monocyte-derived macrophages. We visualized transfer of bacRNA into the cytosol of cells using a recently developed sensitive, non-radioactive but non-toxic method to label RNA in living cells [52] (Fig. 2): 5-ethynyluridine (EU) was shown to be incorporated into RNA transcripts generated by RNA polymerases I, II and III in mammalian cells but not into DNA [52]. *Listeria* were grown in medium containing EU and FITC. The host cells were then infected with labeled bacteria. One or four hours post infection, host cells containing *Listeria* in the cytosol were fixed, permeabilized and incubated with fluorescence dye coupled to a reactive azide group (Alexa594-azide). In this setting, the azide selectively couples to the ethynyl group of EU incorporated into the bacRNA. Whole *Listeria* are labeled green with FITC; RNA is visible as red fluorescence (Alexa594), nuclei are stained by DAPI (blue). As evident from fluorescence images, the cytosol of THP-1 cells was clearly labeled for EU-containing RNA when infected with wild type (wt) *L. monocytogenes* (Fig. 2A left and middle panel) but not when infected with a mutant lacking LLO (hly-) (Fig. 2A right panel) and therefore impaired in its escape from the endosome.

Bacterial RNA accumulated in the cytosol of host cells upon prolonged infection time with wt *L. monocytogenes* (Fig. 2A, left and middle panel). In concordance with findings from THP-1 cells, similar results were obtained with epithelial (A549, Fig. 2B ) and hepatocarcinoma cell lines (HepG2, Fig. 2C). During *Listeria* infection only bacRNA delivered to the cytosol of the host cell could be detected. As direct labeling of RNA from gram negative *E. coli*, which lack a gram positive cell wall, was possible (Fig. S2A), the gram positive cell wall appeared to be the reason for the absence of RNA staining in the bacteria.

To confirm equal incorporation of EU in the RNA of *L. monocytogenes* strains, bacterial RNA of wt and hly^-^ *L. monocytogenes* incubated in EU-containing medium was extracted and labeled with Alexa594-azide in vitro. The data confirm that EU was equally incorporated in wt and hly- *L. monocytogenes* RNA (Fig. S2B). The absence of labeled RNA in the host cell nucleoli, the site of ribosomal RNA production, excluded transfer of EU nucleotides from *L. monocytogenes* with subsequent incorporation into host RNA. In fact, direct labeling of cells in conditioned culture medium led to strong staining of the nucleoli (Fig. S2C). Of note, EU labeling was performed in starvation medium that facilitates the incorporation of EU. Indeed, EU is not quantitatively incorporated in host RNA when cultured in standard culture medium for this short time span (Fig. S2D). Since we cultivated cell lines in standard culture medium during infection, we additionally excluded the possibility that host cell RNA is stained by non-incorporated EU released from *L. monocytogenes*.

From the data we conclude that during infection, significant amounts of bacRNA are transferred into the cytosol of cells where it can be detected by cytosolic immune receptors.

THP-1 cells infected with *L. monocytogenes* lacking the secA2 secretion system, which was recently identified to be responsible for secretion of bacterial RNA of *L*. *monocytogenes*, were not labeled for EU-containing RNA (Fig. S3), suggesting that translocation of bacterial RNA is rather mediated by active secretion than by lysis of bacteria [53].

Type I IFN induction by *L. monocytogenes* in epithelial cells and hepatocytes is triggered by recognition of bacterial RNA.

**Figure 3:**
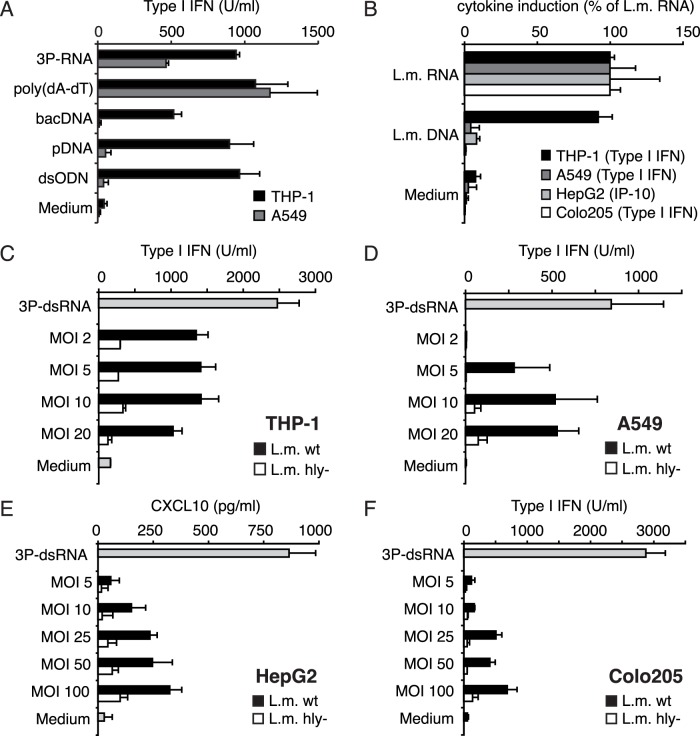
Recognition of bacterial RNA or DNA varies for different cell types. A: THP-1 and A549 cells were transfected with double stranded triphosphorylated RNA (3P-dsRNA), poly(dA-dT), bacterial DNA (bacDNA), plasmid DNA (pDNA) or double stranded 84 mer DNA oligonucleotides (dsODN); B: THP-1, A549, HepG2 and Colo205 cells were transfected with *L. monocytogenes* RNA, *L. monocytogenes* DNA or 3P-dsRNA. Type I IFN (THP-1, A549, Colo205) or CXCL10 (HepG2) production was analyzed 24 hours after stimulation. The relative induction of the indicated cytokine is depicted as percentage of induction by transfected *L. monocytogenes* (L.M.) RNA. C, D, E and F: THP-1, A549, HepG2 and Colo205 cells were infected with wt and hly- *L. monocytogenes* at the indicated MOI. Type I IFN (THP-1, A549, Colo205) or CXCL10 production (HepG2) was analyzed 24 hours after stimulation. Error bars represent s.d.

Work by Ishii et al. [54] suggested that cytosolic recognition of DNA represents an ubiquitous pathway expressed in a wide range of cell lines and cell types. More recent studies exhibited that in human cell lines this refers only to the recognition of AT-rich [23], [55] sequences. Long AT-rich DNA sequences (poly(dA-dT)) were found to be the template of RNA polymerase III (pol III) leading to synthesis of triphosphorylated RNA, the ligand of RIG-I [23], [24]. However, non-AT rich dsDNA molecules in the size range of poly(dA-dT) still induced a type I IFN response in human monocyte-derived dendritic cells (MoDC) in which RIG-I is silenced [23]. This points to two distinct DNA recognition pathways in immune cells: pol III/RIG-I dependent recognition of AT-rich DNA and STING dependent (polIII/RIG-I independent) type I IFN induction by long random DNA. This redundancy in DNA sensing mechanisms complicates the delineation of pattern recognition receptors involved in *L. monocytogenes* sensing. Recent data suggested that the STING dependent pathway functions mainly in immune cells, whereas tumor cell lines such as HEK293 cells only responded to poly(dA-dT) in a pol III/RIG-I dependent fashion [23]. Indeed, we also observed that while THP-1 cells responded to all kinds of transfected DNA including poly(dA-dT), plasmid DNA (pDNA), genomic bacterial DNA and an 84mer double stranded DNA oligonucleotide (dsODN), the lung epithelial cell line A549 only responded to poly(dA-dT) and double stranded 5′ triphosphate RNA (3P-dsRNA) (Fig. 3A). Analogous results were obtained when we used bacRNA or bacDNA derived from *Listeria* (Fig. 3B). Both transfected bacRNA and bacDNA induced equal amounts of type I IFN in monocytic THP-1 cells (Fig. 3B). By contrast, A549, HepG2 (hepatocarcinoma) and Colo205 (colon carinoma) cells were able to sense transfected bacRNA but did not induce a type I IFN response upon transfection of bacDNA (Fig. 3B). We thereby conclude that type I IFN induction by *L. monocytogenes* bacDNA is restricted to immune cells with intact STING- dependent recognition pathways, which are not functional in human non-immune cells. By contrast, non-immune cells are exclusively triggered by bacRNA to induce type I IFN.

Subsequently we tested whether *L. monocytogenes* infection of cell lines, unresponsive to bacDNA, can still induce a type I IFN response. To this end, we infected the indicated cell lines with wild type (wt) or LLO deficient (hly-) *L. monocytogenes* at the MOI shown and assessed the type I IFN secretion (Fig. 3C,D, E, F). THP-1 cells, which can respond to bacDNA, raised a robust type I IFN response upon infection with wt *L. monocytogenes* (Fig. 3C). Strikingly, cell lines derived from non-immune cells lacking a type I IFN response to bacDNA (A549, HepG2, Colo205) also induced substantial amounts of type I IFN or the type I IFN regulated chemokine CXCL10 (HepG2) when infected with wt *L. monocytogenes* (Fig. 3D, E, F). In all analyzed cell lines *L. monocytogenes* induced type I IFN/CXCL10 in a LLO dependent manner, strongly suggesting cytosolic recognition of bacRNA (Fig. 3C, D, E, F). Together these results indicate that bacRNA translocated to the cytosol is the causative agent for *L. monocytogenes-*mediated type I IFN induction in non-immune cells.

### Type I IFN Induction by *L. monocytogenes* Infection Requires RIG-I in Epithelial but not in Monocytic Cells

**Figure 4:**
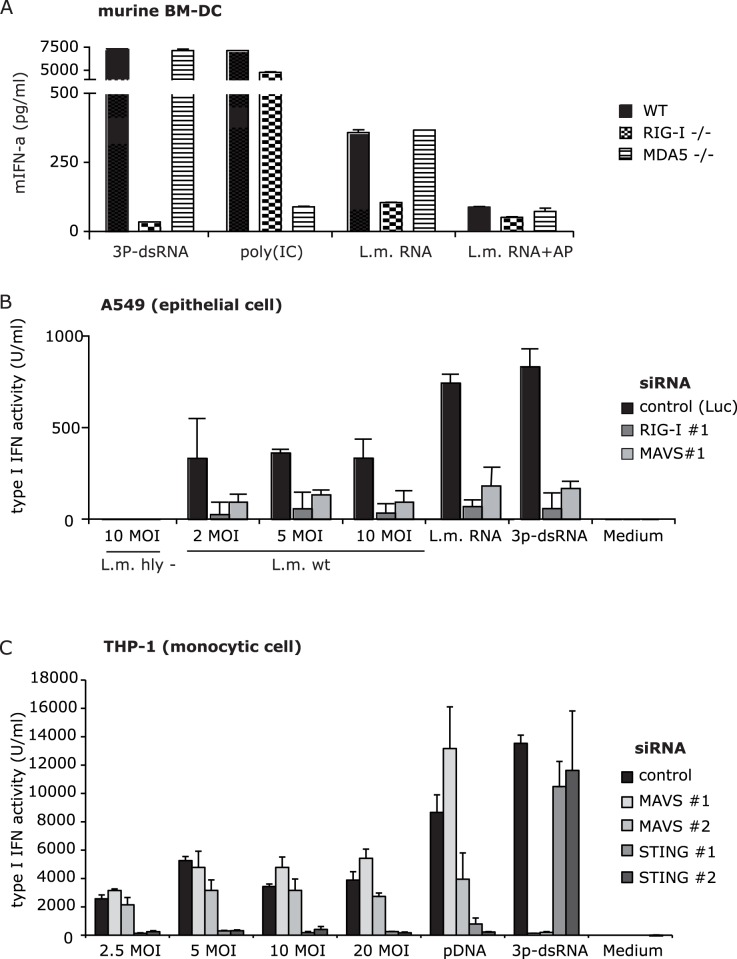
Knockdown of RIG-I abrogates *L. monocytogenes*-induced type I IFN induction in epithelial but not in monocytic cells. A: Murine BM-DCs were transfected with indicated stimuli. One out of two experiments is shown. Murine IFN-α secretion was analyzed 24 hours after stimulation. Error bars represent SEM. B: A549 cells were transfected with siRNA against RIG-I, MAVS or Luciferase (control). Cells were then infected with *L. monocytogenes* or transfected with *L. monocytogenes* RNA (L.m. RNA), *L. monocytogenes* DNA (L.m. RNA) or 3P-dsRNA 48 hours after knock-down. Type I IFN production was analyzed 24 hours after stimulation. B) THP-1 cells were electroporated with control siRNA or siRNAs against MAVS or STING. 72 hours after electroporation, THP-1 cells were infected with hly^-^ or wt *L. monocytogenes* at indicated MOI or transfected with plasmid DNA (pDNA) or RIG-I ligand (3P-dsRNA). Type I IFN production was analyzed 24 hours after stimulation. Relative type I IFN induction was normalized to cells transfected with siRNA against Luciferase and stimulated with pDNA. Error bars represent s.d.

Since epithelial cells were able to respond to transfected bacRNA but not transfected bacDNA, we examined if cytosolic RNA receptors are essential for recognition of bacterial RNA during infection. RIG-I, recognizing 5′triphosphorylated RNAs, is a likely candidate cytosolic RNA sensor for *L. monocytogenes*. To determine the RNA receptor responsible for bacRNA-mediated type I IFN induction we used bone marrow-derived dendritic cells (BM-DC) of RIG-I or MDA5-deficient mice. Indeed, as shown using wild type, RIG-I or MDA5 deficient bone marrow-derived dendritic cells (BM-DC), the *Listeria* RNA-triggered IFN-α induction was exclusively RIG-I dependent (Fig. 4A). Treatment of A549 cells with siRNAs against RIG-I and MAVS abolished the response to the 3P-dsRNA RIG-I ligand (Fig. 4B) 12fold and fourfold, documenting an efficient knock-down of the RIG-I signaling pathway. Accordingly, as anticipated, siRNA mediated knock-down MAVS extensively inhibited type I IFN production of A549 cells during infection with *L. monocytogenes* (Fig. 4B; Fig. S4A). By contrast, in THP-1 cells, which can recognize bacDNA and bacRNA, type I IFN induction did not appear to be influenced by siRNA-mediated knockdown of MAVS during infection with *L. monocytogenes* (Fig. 4C), while the type I IFN response to the RIG-I ligand 3P-dsRNA was efficiently downregulated. The response to DNA was still intact after knock-down of MAVS, excluding the involvement of a polIII/RIG-I stimulatory effect of this DNA type in these cells. Currently, STING is the only known adaptor protein upstream of IRF3 in the DNA recognition signaling pathway and has been shown to be essential for the type I IFN response induced upon *L. monocytogenes* infection [16], [17], [42]. Knockdown of STING strongly inhibited the type I IFN induction response to plasmid DNA(pDNA) in THP-1 cells (Fig. 4C). In contrast to MAVS, knockdown of STING significantly reduced type I IFN induction during *L. monocytogenes* infection of THP-1 cells. We conclude that induction of type I IFN during *Listeria* infection of STING pathway deficient cells is dependent on RIG-I, while the RIG-I pathway is redundant in immune cells such as monocytes, as they possess a STING-dependent pathway and are therefore able to sense c-di-AMP and bacDNA in the cytosol in a pol III/RIG-I independent manner.

## Discussion

RIG-I is considered to be essential for the immune recognition of most negative strand and positive strand RNA viruses (reviewed in [56]). Little is known, however about the involvement of RIG-I and of bacterial RNA in the innate immune recognition of bacteria. Here we found that both the RNA extracted from extracellular and facultative intracellular bacteria induced type I IFN in a 5′-phosphorylation-dependent manner. We analyzed the effect of *L. monocytogenes* infection on immune cells and non-immune cells, both involved in the pathogenesis of *L. monocytogenes*. Infection with wt *L. monocytogenes* led to the induction of type I IFN in all cell lines tested, including monocytic cells and cell lines derived from lung (A549), intestine (Colo205) or liver (HepG2). Using a recently developed sensitive RNA fluorescence labeling technique [52] we demonstrate that during natural infection, the RNA of *L. monocytogenes* indeed gains access to the cytosol of the host cells. Both cytosolic translocation of bacRNA and type I IFN induction were dependent on the presence of the pore forming protein LLO. Although transfer of shRNA from LLO-overexpressing *E. coli* has been reported [57], this is the first time that translocation of endogenous bacterial RNA into the cytosol of the host cell could be visualized.

Previous studies on human cells revealed that LLO is not needed for egress from the vacuole. Paschen et al. [58] observed that in human dendritic cells lack of listeriolysin retarded but did not prevent egress of *L. monocytogenes* from the vacuole. It is tempting to speculate that LLO-deficient Listeria are at least impaired in entering the cytosol, leading to less or retarded secretion of RNA. The fact that secA2-deficient *L. monocytogenes* fails to translocate bacRNA into the cytosol confirm recent findings that RNA is released by secretion and not by lysis of bacteria [53]. Nevertheless, as suggested previously, cytosolic bacterial DNA or c-di-AMP released at the same time as bacRNA could represent an equal or even dominant source of type I IFN induction [13], [59]. However, we found that while transfected bacRNA and bacDNA induced equal amounts of type I IFN in monocytic cells (primary monocytes and THP-1 cells), non-immune cell types were able to sense transfected bacRNA but did not raise a type I IFN response upon transfection of bacDNA, despite a robust response to poly(dA-dT), indicating the absence of functional STING-dependent recognition pathways. The latter finding also excludes an involvement of the pol III/RIG-I dependent pathway of bacDNA detection as suggested by Chu et al. [24] but corroborates data of Monroe et al. [59] for *L. pneumophila* bacterial DNA, which was shown not to trigger the pol III/RIG-I dependent pathway.

Despite translocation of bacRNA into the cytosol, RNAi of RIG-I or MAVS in monocytic cells (THP-1) did not impair type I IFN induction during infection with *L. monocytogenes*. This suggests a redundant role of RIG-I regarding type I IFN induction in monocytes during *L. monocytogenes* infection, as shown for *L. pneumophila* [59]. The minor contribution of the RIG-I/MAVS pathway in monocytic cells may be due to a dominance in concentration or effect of other stimuli released by *L. monocytogenes,* including bacDNA or the recently discovered mediator cyclic diadenosine monophosphate (c-di-AMP) [34].

By contrast, induction of type I IFN during *L. monocytogenes* infection of epithelial cells was nearly completely abolished by RNAi-mediated knockdown of RIG-I or MAVS, implicating that neither DNA nor c-di-AMP plays a role for *L. monocytogenes* mediated type I IFN induction in these cells. Together, we conclude that RIG-I plays a major role in the *L. monocytogenes*-induced secretion of type-I-IFN in cell types lacking functional STING-dependent pathways.

Cytosolic recognition of bacterial RNA is controversial. Mancuso and colleagues [60] showed that phagosomal bacteria, but not cytosolic bacteria, potently induce IFN in conventional dendritic cells by a mechanism that required the RNA-sensing endosomal TLR 7. Although purified transfected *L. monocytogenes* RNA was observed to induce type I IFN [61], previous studies suggested that IFN induction by *L. monocytogenes* occurs in a MAVS-independent, and thereby cytosolic RIG-I independent way [29], [33]. However, in line with our current findings some bacterial RNA species represent a perfect RIG-I target structure: In contrast to eukaryotes, bacteria do not harbor 5′end mRNA cap structures but 5′mono or 5′triphosphate, a major requirement for RIG-I recognition [10]. In *E. coli,* one third of mRNA remains 5′triphosphorylated [39]. Recent studies reported that the 5′phosphorylation status of bacterial mRNA is regulated by the pyrophosphatase RppH [62] regulating mRNA decay [63]. The 5′triphosphate moiety was described to protect mRNA from decay by the bacterial RNase E [63]. Interestingly, the pyrophosphatase RppH was shown to strongly prefer dephosphorylation of single-stranded triphosphorylated 5′nucleotides over base paired ends [62]. This finding was correlated with the fact that bacterial RNAs can be stabilized by a 5′-terminal stem-loop [64], [65]. Therefore the occurrence of 5′base paired triphosphorylated RNA appears to be characteristic for bacteria and represents a pathogen-associated molecular pattern (PAMP). 5′end base paired triphosphorylated RNA was shown to be the ligand for RIG-I [11].

Indeed, studies on recognition of the intracellular bacterium *Legionella pneumophila* exhibited a MAVS-dependent pathway leading to IFN-β stimulation of lung epithelial cells (A549) [66]. More recent studies revealed that crude RNA isolated from *Legionella pneumophila* is recognized in a RIG-I dependent manner when transfected [59]. However, in that study infection with bacteria was examined in murine macrophages, which harbor several type I IFN inducing receptor systems, including direct STING-dependent DNA recognition. This complicates the evaluation of the contribution of RIG-I to the type I IFN response. In macrophages, the RIG-I pathway appeared to play a rather redundant role for immune recognition of intracellular bacteria [59]. Nonetheless, this finding might be cell type specific. So far, the impact of RIG-I during *L. monocytogenes* infection of other cell types than monocytes has not been addressed. *In vivo*, *L. monocytogenes* enters the body by crossing the intestinal barrier into the blood (reviewed in [40]). This involves bacterial invasion and survival within a large variety of non-phagocytic cells [41]. In immune-compromised individuals, multiplication can occur in hepatocytes.

Together, our current data show that RNA of the facultative intracellular bacterium *L. monocytogenes* has quantitative access to the cytosol of the host cell, and that detection of bacterial RNA by the cytosolic immune receptor RIG-I plays a non-redundant role in non-immune cells with impaired DNA/c-di-AMP (STING dependent) recognition pathway such as hepatocytes, a cell type involved in the propagation of bacteria in chronic infection, and colon epithelial cells, a cell type that is critically involved in the enteral route of infection. Interestingly, Li et al. found that RNA of commensal bacteria is recognized in a MAVS-dependent manner and that MAVS in cells of non-hematopoietic origin plays a dominant role in preventing DSS-induced colitis [61].

Considering the fact that mice with a defect in the type I IFN pathway exhibit a strong resistance to *Listeria*-induced pathogenesis, it remains to be determined if the observed RIG-I dependent recognition of bacterial RNA contributes more to the pathogenicity or to the clearance of *Listeria* [67], [68].

## Materials and Methods

### Ethics Statement

The PBMC studies were approved by the local ethics committee (Ethikkommission der Medizinischen Fakultät Bonn) according to the ICH-GCP guidelines. Written informed consent was provided by voluntary blood donors.

The accommodation and care of animals used for experimental and other scientific purposes occurred in the mouse core facility (HET) of the University Hospital Bonn according to the guidelines of the Federation of European Laboratory Animal Science Associations (*FELASA*) and GLP guidelines of the OECD.

### Bacteria

The *L. monocytogenes* wild-type strain EGD and isogenic deletion mutant strains *Δhly, ΔsecA* were cultured in brain-heart infusion (BHI) until they reached log phase and then used for experiments.

### Stimulatory Nucleic Acids

Bacterial RNA was isolated using the Qiagen RNeasy mini kit according to manufacturer’s instructions (Qiagen). Genomic DNA was isolated by lysing the cells with lysozyme and then precipitating the DNA using phenol-chloroform extraction. 3P-dsRNA was generated by *in vitro* transcription as described [11].

### Cell Culture and Stimulation

Human PBMC were isolated from whole human blood of healthy, voluntary donors by Ficoll-Hypaque density gradient centrifugation (Biochrom). PBMCs were cultured in RPMI medium supplemented with 10% FCS and 1% penicillin and streptomycin. Cell lines were cultured in RPMI (THP-1, HepG2) or DMEM (Colo205) medium supplemented with 10% FCS and 1% Penicillin and Streptomycin. For stimulation cells were seeded into 96-well plates. Nucleic acid stimuli were used at a final concentration of 0,8 µg/mL. RNA and DNA stimuli were complexed with Lipofectamine 2000 (Invitrogen). Cell lines were incubated for 2 hours (THP-1) or 4 hours (A549, HepG2 and Colo205) with *L. monocytogenes* and then inactivated using Gentamycin (THP-1) or penicillin (for A549, HepG2 and Colo205). For infection of A549, HepG2 and Colo205 bacteria were pretreated with 10% human AB serum for 30 min at 37°C and washed with PBS.

Secretion of cytokines was measured using ELISA kits supplied by *ebioscience* (human IFN-α) and BD Pharmingen (human CXCL10). Murine IFN-α was determined by sandwich ELISA using standard protein and antibodies from R&D Systems and BIORAD. Human type I IFN activity was quantified by incubation of the type I IFN-sensing reporter cell line HEK-Blue™ (Invivogene) with supernatants of stimulated cells. After 24 h incubation, supernatants were assessed for SEAP activity using substrate pNPP (Sigma) according to the manufacturer’s protocol. RIG-I and MDA5-deficient mice were generated as described [7], [8]. Murine BM-DC were generated by culturing murine bone marrow cells for 7 days with GM-CSF.

### RNAi in Cell Lines

A549 cells in 96 well plates were incubated with 0.1 µg siRNA against RIG-I, MAVS and Luciferase complexed to 1.5 µL Hiperfect (Qiagen; Fig. 4B) or 0.25 µL Lipofectamin2000 (Fig. S4A). Infection or transfection of stimuli into the cells was performed 36 (Fig. S4A) or 48 (Fig. 4B) hours after siRNA transfection. THP-1 cells were electroporated with siRNA 72 hours prior to stimulation. SiRNAs with 3′dTdT were purchased from Biomers (Ulm, Germany) and targeted the following sequences: STING #1: GCAUCAAGGAUCGGGUUU [42]; STING #2: GGUCAUAUUACAUCGGAU; MAVS #1: UUAAAGGAGUUUAUCGAUGUA; MAVS #2: CCCAGAGGAGAAUGAGUAUAA; RIG-I #1: AAGGCUGGUUCCGUGGCUUUU [23]; RIG-I #2: AAGGGAACGAUUCCAUCACU Controls were: Luciferase : CAUAAGGCAAUGAAGAGAUAG; nonsense#1: GAAGUCCUUAACGCGGCAA (Fig. 4C, Fig. S4A).

### Labeling of RNA

*L. monocytogenes* were grown in an overnight culture and then 2 mL of HT-Medium [43] inoculated with 500 µL *L. monocytogenes* suspension. 1 mM EU (Invitrogen) was added and *L. monocytogenes* grown for 4 hours. Bacteria were labeled with 2 mg/mL FITC (Sigma-Aldrich) and washed with 5 µM EDTA/PBS, then resuspended in Opti-MEM prior to infection of host cells [44]. *L. monocytogenes* were used for infection at the appropriate MOIs, then washed off after 1 or 4 hours. Cells were washed with PBS and fixed with 4% PFA in PBS, then treated with Alexa 594 azide and the labeling kit *Click-iT* (Invitrogen) according to manufacturer’s instructions. Briefly, cells were permeabilized with 0.5% Triton X-100 in PBS, then blocked with 1% BSA and washed with PBS. The Click-iT reaction cocktail was assembled and mixed with Alexa 594 azide at 5 µM final concentration. Cells were incubated with the Alexa 594 azide-containing Click-iT reaction cocktail for 30 minutes at room temperature, then washed and treated for fluorescence microscopy.

For labeling of extracted bacterial RNA, overnight wt and hly *L. monocytogenes* cultures were diluted 1∶5 in Fraser Half Medium (FHM). Ethynyl Uridine (EU, Invitrogen) was added to a final concentration of 10 µM and bacteria grown for 4 hours at 37°C. Bacteria RNA was isolated using a modified protocol applied by Toledo-Arana [45]: Bacteria were pelleted and resuspended in solution A (10% w/v glucose, Tris 12.5 mM pH 7.6, 10 mM EDTA). 60 µL 0.5M EDTA and 60 µL of a 10 mg/mL Lysozyme solution were added and bacteria lysed for 2 hours at 37°C. RNA was isolated by standard phenol/chloroform extraction using TRIzol reagent (Invitrogen). RNA was incubated with the Click-iT reaction cocktail containing Alexa Fluor 488 azide. The quantification of EU presence in *Listeria* RNA was performed by measuring the fluorescence of Alexa 488 Fluor-stained RNA from hly- and wt Listeria in a Perkin-Elmer EnVision reader. As a control, equal amounts of RNA from *L. monocytogenes* grown in medium without EU was incubated in the Click-iT reaction cocktail. The background fluorescence of non-EU containing RNA after Click-iT reaction was subtracted from EU containing RNA and normalized on fluorescence of RNA from wt Listeria.

### Fluorescence Microscopy

Freshly trypsinized and washed A549 or HepG2 were seeded at the appropriate concentrations onto uncoated coverslips, then left to adhere for 3 hours at 37°C. FITC-tagged and EU-labeled *L. monocytogenes* were then added to the cells and left for infection for the indicated duration. After fixation and treatment with the Click-iT reaction cocktail, cells were washed with PBS and counterstained with DAPI (Thermo scientific). THP-1 cells were infected and stained in solution and resuspended in mounting medium. Cells were then visualized using a fluorescence microscope (Zeiss).

## Supporting Information

Figure S1 **Effect of chloroquin incubation.** Human PBMC were preincubated with chloroquine or left untreated and transfected with indicated nucleic acids. IFN-α production was analyzed 24 hours after stimulation.
(EPS)

Figure S2 **Specificity of Click RNA labeling.** A: *E. coli* bacteria were incubated with EU during log phase growth and EU labeled RNA stained with Alexa Fluor 594 azide. B: Whole RNA was extracted from wild type (wt) and LLO deficient (hly-) *L. monocytogenes* bacteria grown in EU-containing medium and coupled to Alexa Fluor 488 azide. Fluorescence of EU/Alexa Fluor 488 azide-labeled RNA was measured in solution. Background fluorescence of non-EU containing RNA after the Click-iT reaction was subtracted from EU containing RNA and normalized on fluorescence of RNA from wt *L. monocytogenes*. Mean values+s.d. (% of wt RNA) of three independent experiments are shown. THP-1 cells were grown for 12 hrs in starvation medium (RPMI without FCS) (C) or in regular medium (D) and then incubated for 4 hrs with equal concentrations of EU. Cells were fixed and EU-labeled RNA counterstained with Alexa 594.
(EPS)

Figure S3 **Release of bacterial RNA into the cytosol depends on the SecA2 secretion system.** THP-1 were infected with FITC-tagged and EU-labeled wild type (L.m.wt; left column) and SecA2 deficient (L.m. SecA-; left column) *L. monocytogenes* for two hours. Cells were then fixed, stained with Alexa594-azide and counterstained with DAPI. Whole *L. monocytogenes* are labeled green with FITC and RNA is visible as red fluorescence (Alexa594), nuclei are stained by DAPI (blue). Three examples per condition are shown. As determined by counting of single stained bacteria in cells (100 cells per slide were counted) the average bacterial load was 0.5(wt) and 0.5(SecA-) bacteria per cell. One representative experiment out of three is shown.
(EPS)

Figure S4 **A: A549 cells were transfected with siRNA against RIG-I, MAVS or a control sequence.** Cells were then infected with *L. monocytogenes* 40 hours after knock-down. Type I IFN production was analyzed 24 hours after stimulation. B: mRNA expression levels of RIG-I (left panel) or MAVS (right panel) normalized on GAPDH expression, 40 h after transfection of siRNAs into A549 cells. C: mRNA expression levels of STING (left panel) or MAVS (right panel) normalized of GAPDH expression, 72 h after electroporation. Error bars represent s.d.
(EPS)
